# Genetic markers in Andean *Puya* species (Bromeliaceae) with implications on plastome evolution and phylogeny

**DOI:** 10.1002/ece3.9159

**Published:** 2022-07-29

**Authors:** Lu Liu, Yu‐Qu Zhang, Liscely Tumi, Mery L. Suni, Mónica Arakaki, Kevin S. Burgess, Xue‐Jun Ge

**Affiliations:** ^1^ Key Laboratory of Plant Resources Conservation and Sustainable Utilization, and Guangdong Provincial Key Laboratory of Applied Botany, South China Botanical Garden Chinese Academy of Sciences Guangzhou China; ^2^ University of Chinese Academy of Sciences Beijing China; ^3^ College of Pharmacy Shaanxi University of Chinese Medicine Xi'an China; ^4^ Facultad de Ciencias Biológicas Universidad Nacional Mayor de San Marcos Lima Peru; ^5^ Department of Biology, Columbus State University University System of Georgia Columbus Georgia USA; ^6^ Center of Conservation Biology, Core Botanical Gardens Chinese Academy of Sciences Guangzhou China

**Keywords:** divergence time estimation, genome feature, hypervariable region, phylogenetic reconstruction, plastome, *Puya*

## Abstract

The Andean plant endemic *Puya* is a striking example of recent and rapid diversification from central Chile to the northern Andes, tracking mountain uplift. This study generated 12 complete plastomes representing nine *Puya* species and compared them to five published plastomes for their features, genomic evolution, and phylogeny. The total size of the *Puya* plastomes ranged from 159,542 to 159,839 bp with 37.3%–37.4% GC content. The *Puya* plastomes were highly conserved in organization and structure with a typical quadripartite genome structure. Each of the 17 consensus plastomes harbored 133 genes, including 87 protein‐coding genes, 38 tRNA (transfer RNA) genes, and eight rRNA (ribosomal RNA) genes; we found 69–78 tandem repeats, 45–60 SSRs (simple sequence repeats), and 8–22 repeat structures among 13 species. Four protein‐coding genes were identified under positive site‐specific selection in *Puya*. The complete plastomes and hypervariable regions collectively provided pronounced species discrimination in *Puya* and a practical tool for future phylogenetic studies. The reconstructed phylogeny and estimated divergence time for the lineage suggest that the diversification of *Puya* is related to Andean orogeny and Pleistocene climatic oscillations. This study provides plastome resources for species delimitation and novel phylogenetic and biogeographic studies.

## Graphical abstract

Comparative analyses revealed plastome features, genomic evolution, divergence time, and phylogenetic relationships of the genus *Puya*. Plastomes have great potential for phylogenetic recontraction and species discrimination of *Puya*.

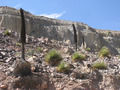

## 1 INTRODUCTION

**FIGURE 1:**
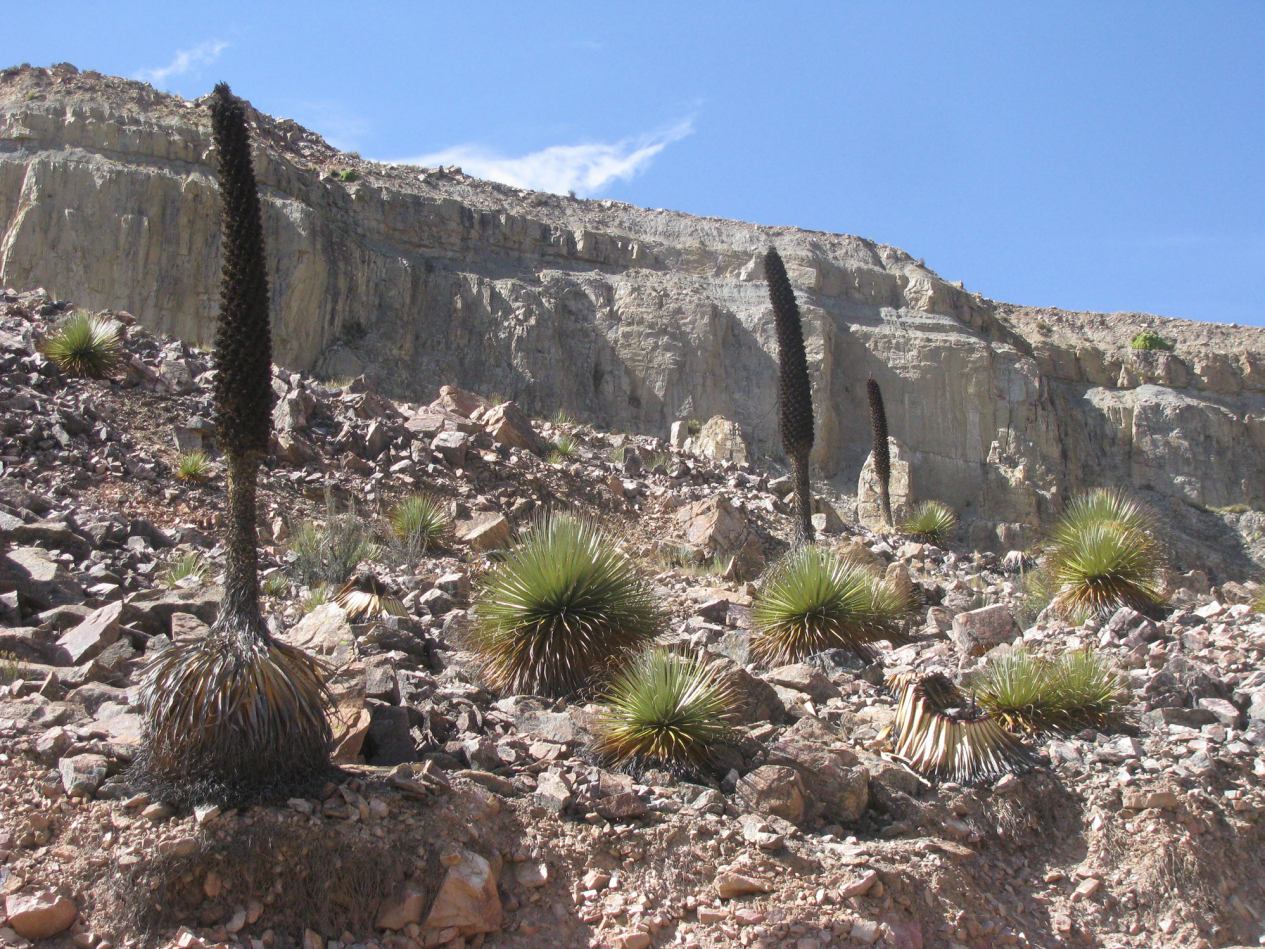
Morphological characteristics of *Puya raimondii*.

*Puya* Molina (Puyoideae, Bromeliaceae) is a characteristic and typical genus of humid or dry mountain ecosystems in the Andes (Luteyn et al., 1999), with 229 species (WCVP, 2021), some of them narrow endemics, distributed from Venezuela to northern Argentina (Smith, 1988). The genus *Puya* are perennial rosette‐forming herbs, terrestrial, sometimes on rocks, frequently flowering several meters high (specific diagnostic can be found in Appendix S1, and one species is shown in Figure 1). Their leaf blades are narrowly triangular and always have coarsely spinose‐serrate margin. The inflorescences are erect, simple, or paniculate, usually pedunculate (Manzanares, 2020; Smith & Downs, 1974). *Puya* is traditionally divided into two subgenera, the subgenus *Puya*, containing eight species (*P. alpestris*, *P. berteroniana*, *P. boliviensis*, *P. castellanosii*, *P. chilensis*, *P. gilmartiniae*, *P. raimondii*, *P. weddelliana*), and the subgenus *Puyopsis*, containing the remainder of the species (Hornung‐Leoni & Sosa, 2008; Smith & Downs, 1974). The subgenus *Puya* can be identified by sterile flowers at the apex of inflorescences, fertile in *Puyopsis* (Manzanares, 2020; Smith & Downs, 1974) and displays remarkable morphological variation, with monocarpic to polycarpic taxa, short to tall stems, and terrestrial to lithophytic ecotypes (Manzanares, 2020). The evolutionary history of the genus, as revealed by phylogenetic and distributional data (Givnish et al., 2011; Jabaily & Sytsma, 2010), makes it an ideal model for the study of rapid speciation in the high‐elevation Andes.

The phylogenetic relationships of Andean *Puya* have not been fully resolved. The first molecular phylogenetic study of *Puya*, based on three plastid sequences and one single‐copy nuclear DNA sequence, supported the monophyly of *Puya*, dividing the genus into two major clades, “Core *Puya*” and “Chilean *Puya*” (Jabaily & Sytsma, 2010). However, the level of informative sequence variation for the “Core *Puya*” clade was too low to resolve species relationships. The same authors (Jabaily & Sytsma, 2013) later analyzed 885 amplified fragment length polymorphism (AFLP) sequences for 75 taxa and the results clustered *Puya* species into two major well‐supported clades corresponding to “Core *Puya*” and “Blue *Puya*” (Chilean species with blue flowers); however, species relationships were also weakly supported.

Genome skimming is a straightforward next‐generation sequencing approach to obtain genetic sequences of the complete plastome for plant species (Dodsworth, 2015). In recent years, plastomes have become a versatile tool for significantly increasing resolution at low taxonomic levels in plant phylogenetic and phylogeographic analyses (Li et al., 2015; Parks et al., 2009). To test the efficacy of plastome sequences for resolving phylogenetic relationships within *Puya*, we generated 12 new *Puya* plastomes representing nine species and compared them to five published *Puya* accessions. Here, we address the following objectives: (1) identify particular features, structures, repeat regions, and positive selection sites of plastomes; (2) assess the utility of plastomes for phylogenetic reconstruction within *Puya*; (3) screen potential DNA barcodes for species discrimination; and (4) estimate divergence times of major lineages of *Puya*.

## 2 MATERIALS AND METHODS

### 2.1 Plant materials and DNA sequencing

A total of 12 accessions representing nine species of *Puya* from high‐elevation Andes were sampled, including *P. nitida*, *P. santosii*, *P. goudotiana*, *P. hamata*, *P. ferruginea*, *P. macropoda*, *P. macrura*, *P. hutchisonii*, and *P. raimondii* (Table 1). Raw sequence data for four *Puya* species were obtained from the NCBI‐SRA (National Center for Biotechnology Information, Sequence Read Archive) database: *P. alpestris*: SRR13700326, *P. coerulea*: SRR9846915, *P. hutchisonii*: SRR10023782, and *P. laxa*: SRR13700325 (Table 1). Additionally, the plastome of *P. mirabilis* was downloaded from GenBank (accession number: NC_045380.1). A total of 17 accessions, representing 13 species of *Puya*, were used in this study.

**TABLE 1:** Characteristics of *Puya* and two other bromeliad plastomes. Sequences with * were downloaded from NCBI

Species	Collector	Country	Elevation (m)	Collection number	Total reads (Mb)	Q30 (%)	Total length (bp)	LSC length (bp)	IR length (bp)	SSC length (bp)	CDS length (bp)	Number of codons	GC content (%)	GenBank accession number	SRA accession number
*P. nitida*	Xue‐Jun Ge	Colombia	3500	ge19046	24.90	89.60	159,799	87,712	26,750	18,587	80,499	26,746	37.30	OL639018	SRR17069090
*P. santosii*	Xue‐Jun Ge	Colombia	3650	ge19047	25.27	89.87	159,752	87,665	26,750	18,587	80,499	26,746	37.30	OL639022	SRR17069066
*P. goudotiana*	Xue‐Jun Ge	Colombia	3440	ge19043	20.51	88.41	159,757	87,669	26,750	18,588	80,499	26,746	37.30	OL639023	SRR17069065
*P. hamata*	Xue‐Jun Ge	Ecuador	3330	ge19035	25.04	89.19	159,772	87,678	26,750	18,594	80,499	26,746	37.30	MZ403751	SRR17050391
*P. ferruginea*	Xue‐Jun Ge	Peru	470	170,510	22.13	90.41	159,713	87,642	26,745	18,581	80,529	26,756	37.30	OL639024	SRR17069064
*P. macropoda*	Yu‐Qu Zhang	Peru	3700	zhpu34	21.34	90.32	159,828	87,733	26,750	18,595	80,538	26,759	37.30	OL639027	SRR17069063
*P. macropoda*	Yu‐Qu Zhang	Peru	3700	zhpu36	20.00	90.41	159,828	87,733	26,750	18,595	80,538	26,759	37.30	OL639028	SRR17069091
*P. macrura*	Yu‐Qu Zhang	Peru	3200	zhpu30	21.59	95.48	159,826	87,732	26,750	18,594	80,538	26,759	37.30	OL639025	SRR17069062
*P. macrura*	Yu‐Qu Zhang	Peru	3200	zhpu31	20.00	95.16	159,826	87,732	26,750	18,594	80,538	26,759	37.30	OL639019	SRR17069060
*P. hutchisonii*	Yu‐Qu Zhang	Peru	4140	zhpu20	21.70	91.46	159,839	87,742	26,750	18,597	80,538	26,759	37.30	OL639026	SRR17069061
*P. raimondii*	Liscely Tumi	Peru	4150	Ch2	20.00	89.10	159,785	87,719	26,738	18,590	80,514	26,751	37.30	OL639020	SRR17069059
*P. raimondii*	Giovana Cesar	Peru	4070	J16	20.00	87.53	159,810	87,719	26,750	18,591	80,538	26,759	37.30	OL639021	SRR17069058
*P. laxa*		Bolivia					159,542	87,459	26,750	18,583	80,538	26,759	37.40		SRR13700325*
*P. alpestris*		Chile					159,607	87,817	26,779	18,232	80,664	26,801	37.40		SRR13700326*
*P. coerulea*		Chile					159,834	88,057	26,773	18,231	80,652	26,797	37.40		SRR9846915*
*P. hutchisonii*		Peru					159,835	87,739	26,750	18,596	80,538	26,759	37.30		SRR10023782*
*P. mirabilis*							159,829	87,800	26,750	18,529	80,532	26,757	37.30	NC_045380.1*	
*Ochagavia elegans*							158,163	86,453	26,754	18,202	80,526		37.50	NC_045385.1*	
*Ananas comosus*							159,636	87,466	26,774	18,622	80,565		37.40	NC_026220.1*	

Total genomic DNA was extracted from silica‐dried leaves using the CTAB (cetyltrimethylammonium bromide) method (Doyle & Doyle, 1987). The library construction and sequencing were executed by the Beijing Genomics Institute (BGI, Wuhan, China). Covaris was used to randomly fragment 1 μg of genomic DNA, followed by fragment selection with an average size of 200–400 bp, PCR amplification, and purification using the Agencourt AMPure XP‐Medium kit. Single‐strand circular DNAs were generated as the final library. The library with concentration greater than 5 ng/μl was qualified by the Agilent Technologies 2100 bioanalyzer (Agilent DNA 1000 Reagents). Libraries that passed this QC step were sequenced by the BGISEQ‐500 platform. About 3 Gb raw data were obtained for each sample, and the generated 150 bp paired‐end (PE) reads were evaluated by FastQC v0.11.9 (https://www.bioinformatics.babraham.ac.uk/projects/fastqc/) and trimmed to remove adaptors using Trimmomatic v0.39 (Bolger et al., 2014) with the following parameters: ILLUMINACLIP:TruSeq3‐PE.fa:2:30:10:8true, LEADING:3, TRAILING:3, SLIDINGWINDOW:4:15, and MINLEN:36. After removing low‐quality reads and the adaptor sequences, clean reads were obtained for each sample.

### 2.2 Plastome assembly and annotation

NOVOPlasty v4.3 (Dierckxsens et al., 2017) was used to assemble the plastomes based on the following settings: Genome range = 130,000–170,000 bp and K‐mer = 39. *Puya mirabilis* (NC_045380.1) was used as the reference plastome. We reassembled the plastomes using GetOrganelle v1.7.5 (Jin et al., 2020); settings for maximum extension rounds = 10 and target organelle genome type = embplant_pt. The final plastomes were annotated using GeSeq (https://chlorobox.mpimp‐golm.mpg.de/geseq.html; Tillich et al., 2017) and CPGAVAS2 (http://47.90.241.85:16019/analyzer/annotate; Shi et al., 2019). They were then manually corrected using Geneious v7.1.4 (Kearse et al., 2012) by comparison to the plastome of *P. mirabilis* (NC_045380.1). Organellar Genome Draw (Greiner et al., 2019) was used to illustrate circular genome maps.

Twelve newly assembled plastomes were deposited in NCBI‐GenBank (Table 1). Codon frequency and relative synonymous codon usage (RSCU) were calculated based on Geneious (Kearse et al., 2012) statistics of protein‐coding genes. The expansion and contraction of inverted repeat (IR) regions at junction sites, including LSC/IRb (the junction of large single‐copy and inverted repeat b), SSC/IRb (small single‐copy and inverted repeat b), SSC/IRa (small single‐copy and inverted repeat a), and LSC/IRa (large single‐copy and inverted repeat a), were analyzed using IRscope (Amiryousefi et al., 2018).

### 2.3 Repeat analyses

A tandem repeat in DNA is composed of two or more adjacent, approximate copies of a pattern of nucleotides. Tandem Repeats Finder v4.09 (Benson, 1999) was used to detect tandem repeats, setting the minimum alignment score to 80. Simple sequence repeats (SSRs) were examined by Perl script MicroSAtellite (MISA) (https://webblast.ipk‐gatersleben.de/misa/; Beier et al., 2017) with the following parameters: minimum SSR motif length of 10 bp and repeat length of mono‐10, di‐6, tri‐5, tetra‐5, penta‐5, and hexa‐5. The maximum size of interruptions allowed between two different SSRs in a compound SSR was 100 bp. We also identified various types of dispersed repeats using REPuter (https://bibiserv.cebitec.uni‐bielefeld.de/reputer; Kurtz et al., 2021), setting the minimum repeat size of 30 bp, the maximum size of 100 bp, and hamming distance of 3. Repeats were classified into the following groups: (1) forward or direct repeats (F); (2) repeats found in reverse orientation (R); (3) palindromic repeats forming hairpin loops in their structure (P); and (4) repeats found in reverse complement orientation (C). Because REPuter overestimates the number of repeats, those with less than 30 bp in length and a redundant output were manually filtered.

### 2.4 Variation analyses

The plastome sequences were aligned using the default option implemented in MAFFT v7.467 (Katoh & Standley, 2013). Using DnaSP v6.12.03 (Rozas et al., 2017), 17 complete *Puya* plastomes were compared to calculate DNA polymorphisms. The plastome's nucleotide diversity was estimated with a step of 200 bp and a window length of 800 bp. The CODEML program of PAML v4.9j (Yang, 2007) was used to detect positive selection. Protein‐coding genes were extracted and aligned in Geneious (Kearse et al., 2012), stop codons were removed manually, and the aligned sequences were converted to PAML format. The model M0 with F3X4 coding frequency was used to calculate the ratio of nonsynonymous and synonymous sites (dN/dS) of each protein‐coding gene among 17 *Puya* plastomes. Site models (M0, M1a, M2a, M3, M7, M8, and M8a) with F3X4 coding frequency were implemented using EasyCodeML (Gao et al., 2019) to detect signatures of positive selection on each gene. The likelihood ratio test (LRT) and the Bayes Empirical Bayes (BEB) method were used to identify sites under positive selection. Gene sites with a dN/dS > 1, *p* < .05 and posterior probability >.95 were considered as putatively selected.

### 2.5 Phylogenetic analyses

To evaluate the efficacy of plastome data for phylogenetic tree reconstruction, we assembled datasets that included sequences from 13 *Puya* species, 12 plastome assemblies generated here, plus five published accessions. These datasets include: (1) complete plastome sequences (including LSC, SSC, and IRb regions), (2) three standard DNA barcode regions (*rbcL* + *matK* + *trnH*‐*psbA*), and (3) the hypervariable regions inferred from DnaSP (Rozas et al., 2017). *Ochagavia elegans* (NC_045385.1), from the sister clade of Bromelioideae, was used as an outgroup.

Based on aligned sequences, jModelTest v2.1.10 (Darriba et al., 2012; Guindon et al., 2003) was used to infer the most appropriate model of nucleotide substitution according to the Akaike information criterion (AIC; Akaike, 1974). As shown in Appendix S2, the GTR + G + I model is the best fit for the plastome phylogenetic reconstruction, while the GTR + G model is the best fit for the standard barcodes and hypervariable regions. MrBayes v3.2.7a (Ronquist et al., 2012) was used to perform Bayesian inference (BI) of the phylogeny based on the best‐fit model and the Markov Chain Monte Carlo (MCMC) method (Drummond et al., 2002) with 20,000,000 samplings every 1000 generations. The maximum likelihood (ML) phylogenetic tree was generated using RAxML v8.2.11 (Stamatakis, 2014) with the best‐fit model and 1000 replicates. The phylogenetic trees were viewed and edited using FigTree v1.4.4 (http://tree.bio.ed.ac.uk/software/figtree) and iTOL v5 (Letunic & Bork, 2021).

### 2.6 Divergence time estimation

Based on the phylogenetic analyses of the plastomes of 13 *Puya* species, divergence time estimations were inferred using BEAST2 (Bouckaert et al., 2019). To avoid biased results by using a single species as an outgroup, we included *Ananas comosus* (NC_026220.1) from the sister subfamily Bromelioideae as an outgroup together with *O. elegans* (NC_045385.1). BEAUti2, within the BEAST2 package, was used for generating BEAST2 XML configuration files. Taxa were grouped into Clades I to III, and outgroup, according to phylogenetic estimations from MrBayes and RAxML (section 2.5). Monophyly was enforced on these groups during the BEAST runs. A secondary calibration point was employed because of the absence of reliable fossil records for Bromeliaceae. According to Givnish et al. (2011), ancestral *Puya* diverged from the ancestral Bromelioideae ca. 10.1 Ma (±1 standard deviation, SD). Thus, we used a normal distribution with a mean age of 10.1 Ma (SD = 1) to calibrate the root age. The divergence time estimation was run under a GTR substitution model along with a gamma site model inferred from jModelTest (section 2.5), a relaxed clock log‐normal distribution (Drummond et al., 2006), and a Yule process tree prior (Yule, 1925). The MCMC chains (Drummond et al., 2002) were conducted with chain lengths set to 100,000,000 and sampling frequencies to 10,000. The convergence of output log files was analyzed using Tracer v1.5, and runs were continued until the effective sample sizes were >200. The maximum clade credibility tree with median heights was generated by TreeAnnotator in the BEAST package, with the initial 10% trees removed as burn‐in. The final tree was edited in FigTree v1.4.4 (http://github.com/rambaut/figtree/).

## 3 RESULTS

### 3.1 Plastome features

**FIGURE 2:**
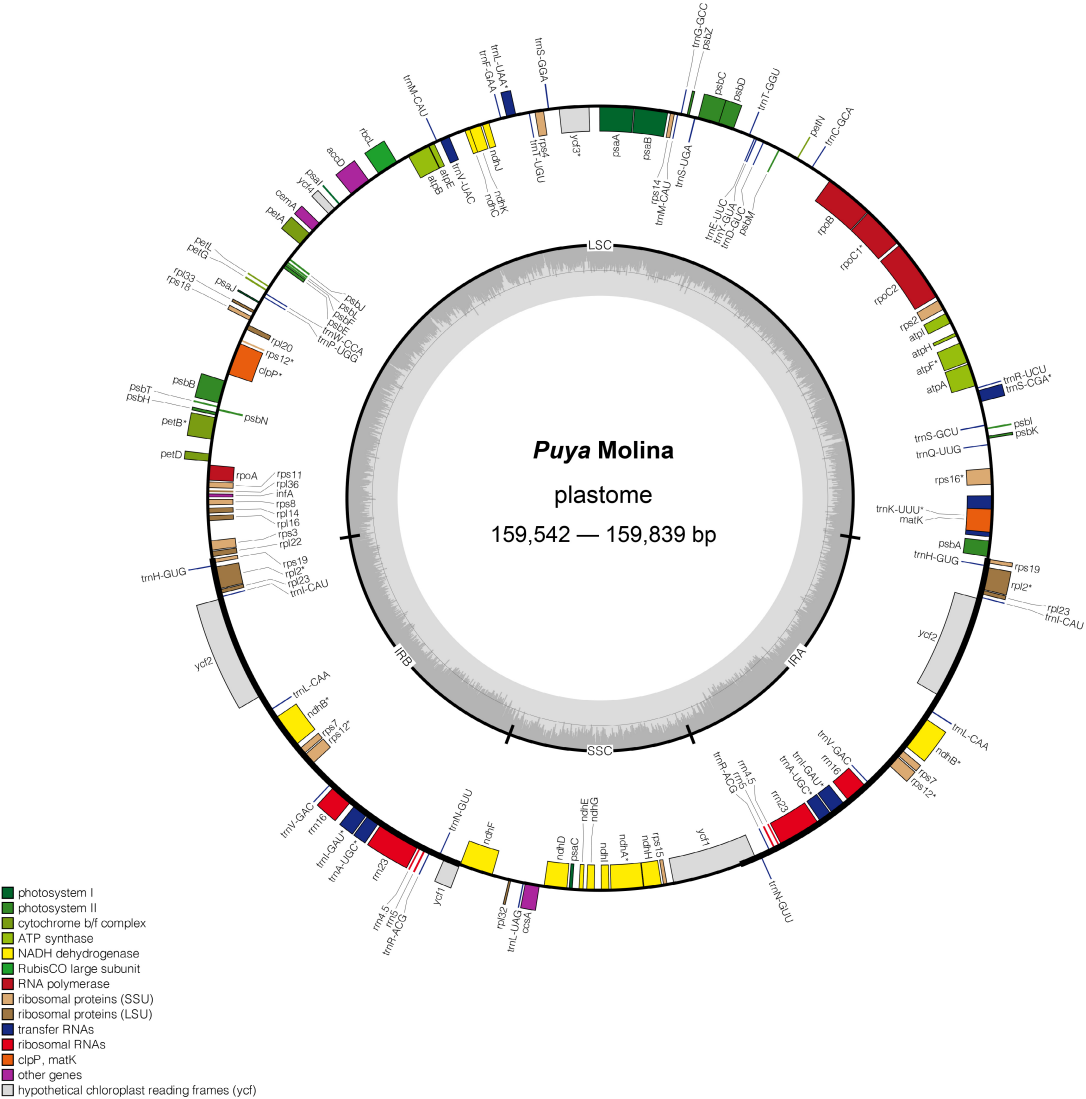
Gene map of the complete plastome of *Puya*. The figure shows a circular representation of the *Puya* plastome with structural organization of the gene content ring which was color coded based on its functional category. The dark gray lines in the innermost circle denotes the GC content across the genome. The genes that were transcribed counter‐clockwise and clockwise were at the outer and inner ring, respectively.

**FIGURE 3:**
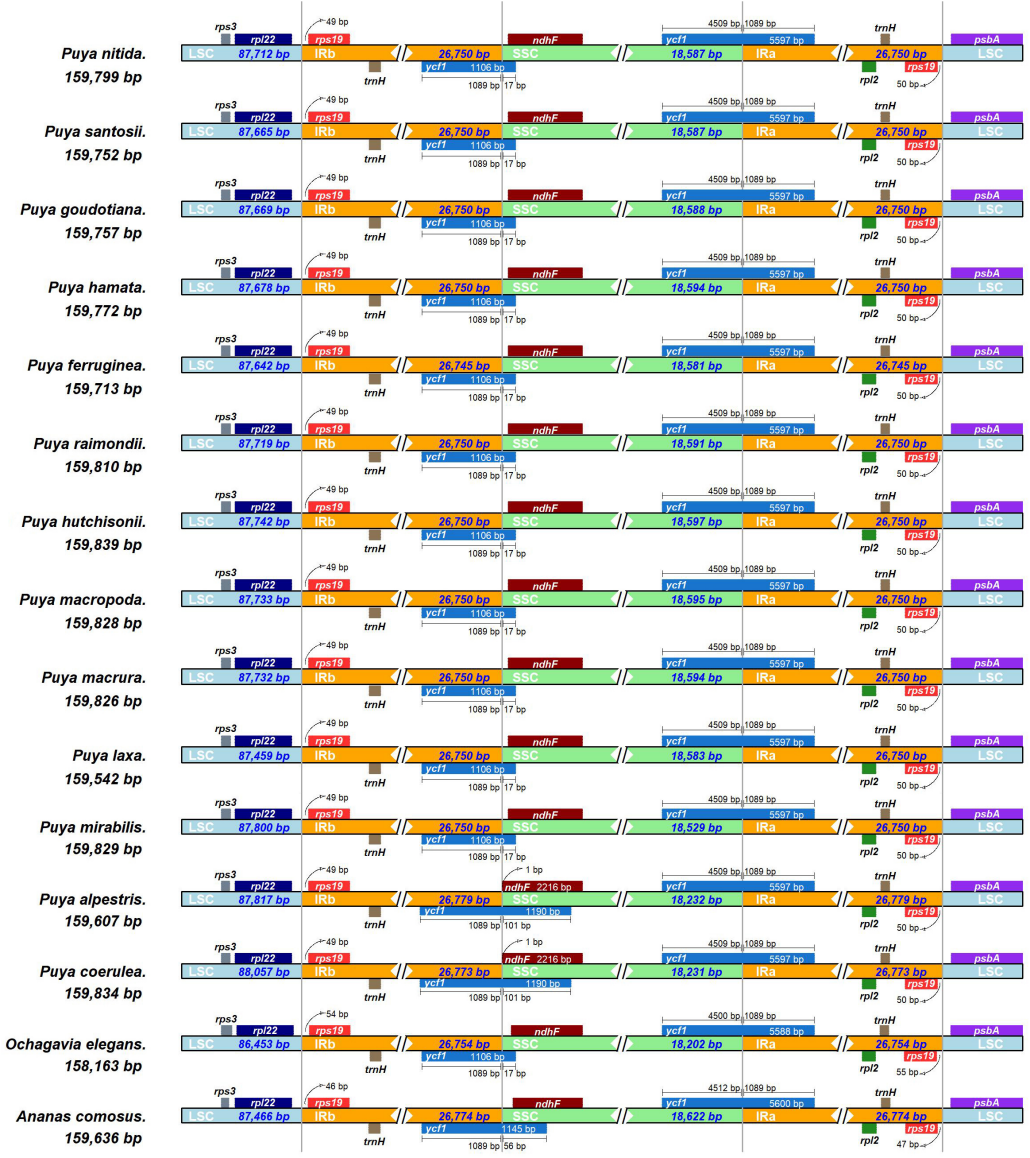
Comparison of the large single copy (LSC), small single copy (SSC), and inverted repeat (IR) borders of 13 *Puya* species and two other bromeliad plastomes.

Overall, 20,000,000–25,268,314 PE 150 bp reads were obtained from 12 newly generated accessions (Table 1). The Q30 ranged from 87.53% to 95.48%. The 12 new plastomes generated by NOVOPlasty and GetOrganelle were identical. The sizes of complete plastomes for 17 *Puya* accessions ranged from 159,542 bp (*P. laxa*) to 159,839 bp (*P. hutchisonii*; Table 1; Figure 2). The *Puya* plastomes had a typical quadripartite genome structure, including an LSC region (87,459–88,057 bp) and an SSC region (18,231–18,597 bp), separated by two IR regions (26,738–26,779 bp; Table 1; Figure 3). The GC content consistently was 37.3%–37.4% in the complete plastome, 42.7% in each IR region, 35.2%–35.4% in the LSC, and 31.3%–31.6% in the SSC region.

The plastome sequences of 17 *Puya* accessions were aligned and annotated using reference‐based (*P. mirabilis*) and de novo method. The results showed that the plastomes were highly conserved within *Puya*. Also, no rearrangement events were detected among the *Puya* species. All plastomes encode 133 genes, including 87 protein‐coding genes, 38 tRNA (transfer RNA) genes, and eight rRNA (ribosomal RNA) genes, with identical gene order (Figure 2; Table 2). Among these genes, eight protein‐coding genes (*rps19*, *rpl2*, *rpl23*, *ycf2*, *ndhB*, *rps7*, *rps12*, *ycf1*), eight tRNA genes (*trnH* ^*GUG*^, *trnI* ^*CAU*^, *trnL* ^*CAA*^, *trnV* ^*GAC*^, *trnI* ^*GAU*^, *trnA* ^*UGC*^, *trnR* ^*ACG*^, *trnN* ^*GUU*^), and four rRNA genes (*rrn16*, *rrn23*, *rrn4.5*, *rrn5*) are duplicated in the IR regions, while 71 protein‐coding genes and 22 tRNA genes are unique in the LSC/SSC region (Figure 2; Table 2). Both SSC/IRb and SSC/IRa in the *Puya* plastome are positioned within the coding region of *ycf1* (with 1089 bp located at IRb and IRa) (Figure 3). The intergenic *rpl22*‐*rps19* is in the LSC/IRb, and the intergenic *rps19*‐*psbA* is in the LSC/IRa (Figure 3).

**TABLE 2:** List of genes in the plastome of *Puya*

Category for genes	Group of genes	Name of genes	Count
Genes for phytosynthesis	Photosystem I	*psaA, psaB, psaC, psaI, psaJ*	5
	Photosystem II	*psbA, psbB, psbC, psbD, psbE, psbF, psbH, psbI, psbJ, psbK, psbL, psbM, psbN, psbT, psbZ*	15
	ATP synthase	*atpA, atpB, atpE, atpF, atpH, atpI*	6
	NADH dehydrogenase	*ndhA, ndhB* (×2)*, ndhC, ndhD, ndhE, ndhF, ndhG, ndhH, ndhI, ndhJ, ndhK*	12
	Cytochrome b/f complex	*petA, petB, petD, petG, petL, petN*	6
	Rubisco large subunit	*rbcL*	1
Self‐replication	Ribosomal proteins (LSU)	*rpl2* (×2)*, rpl14, rpl16, rpl20, rpl22, rpl23* (×2)*, rpl32, rpl33, rpl36*	11
	Ribosomal proteins (SSU)	*rps2, rps3, rps4, rps7* (×2)*, rps8, rps11, rps12* (×2)*, rps14, rps15, rps16, rps18, rps19* (×2)	15
	RNA polymerase	*rpoA, rpoB, rpoC1, rpoC2*	4
	Ribosomal RNAs	*rrn4.5* (×2)*, rrn5* (×2)*, rrn16* (×2)*, rrn23* (×2)	8
	Transfer RNAs	*trnA* ^*UGC*^ (×2)*, trnC* ^*GCA*^ *, trnD* ^*GUC*^ *, trnE* ^*UUC*^ *, trnF* ^*GAA*^ *, trnM* ^*CAU*^ *, trnG* ^*GCC*^ *, trnH* ^*GUG*^ (×2)*, trnI* ^*CAU*^ (×2)*, trnI* ^*GAU*^ (×2)*, trnK* ^*UUU*^ *, trnL* ^*CAA*^ (×2)*, trnL* ^*UAA*^ *, trnL* ^*UAG*^ *, trnM* ^*CAU*^ *, trnN* ^*GUU*^ (×2)*, trnP* ^*UGG*^ *, trnQ* ^*UUG*^ *, trnR* ^*ACG*^ (×2)*, trnR* ^*UCU*^ *, trnS* ^*CGA*^ *, trnS* ^*GCU*^ *, trnS* ^*GGA*^ *, trnS* ^*UGA*^ *, trnT* ^*GGU*^ *, trnT* ^*UGU*^ *, trnV* ^*GAC*^ (×2)*, trnV* ^*UAC*^ *, trnW* ^*CCA*^ *, trnY* ^*GUA*^	38
Other genes	Subunit of acetyl‐CoA‐carboxylase	*accD*	1
	Cytochrome biogenesis protein	*ccsA*	1
	Inner envelope membrane protein	*cemA*	1
	ATP‐dependent protease	*clpP*	1
	Translation initiation factor	*infA*	1
	Maturase	*matK*	1
Genes of unknown function	Hypothetical chloroplast reading frames	*ycf1* (×2)*, ycf2* (×2)*, ycf3, ycf4*	6

**FIGURE 4:**
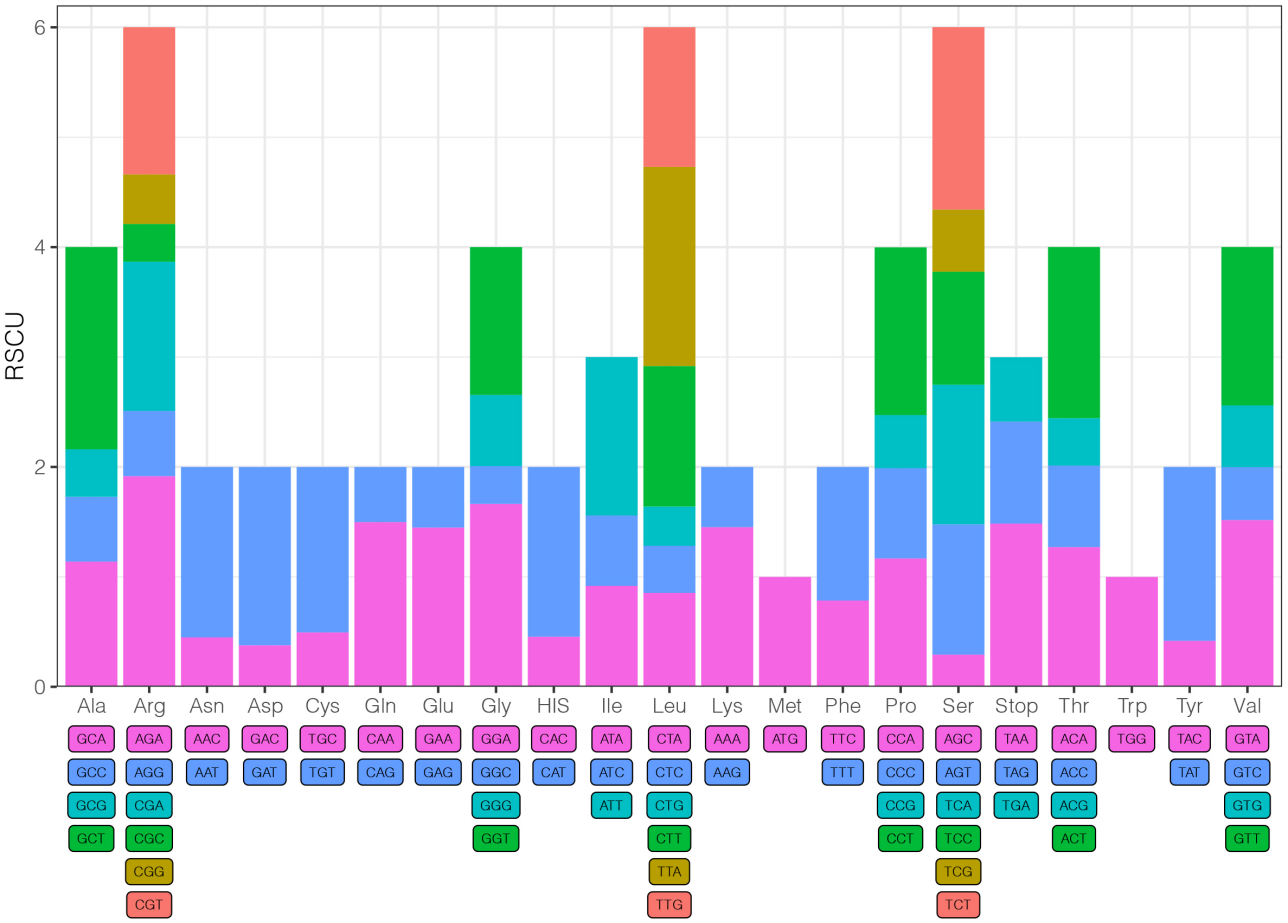
The relative synonymous codon usage (RSCU) of 17 studied *Puya* accessions calculated based on protein‐coding genes. *X*‐axis: Amino acid encoded by different codons; *Y*‐axis: Average value of RSCU of 17 accessions. Color of the histogram is corresponding to the color of codons.

The total length of CDSs (protein‐coding sequences) ranged from 80,499 bp to 80,664 bp, comprising 87 protein‐coding genes and 26,746–26,801 codons, excluding the stop codons (Table 1). The most and the least prevalent amino acids coded were leucine (10.3%) and cysteine (1.2%), respectively (Appendix S3). Meanwhile, the third codon used for every amino acid was biased towards A and T in the *Puya* plastomes (Figure 4), corresponding to many other land plants (Duan et al., 2021; Liu et al., 2020; Ravi et al., 2007).

### 3.2 Plastome repeats

We identified 69–78 tandem repeats, ranging from 3 to 59 bp (Appendix S4). The most common size was 10–19 bp, primarily located in the LSC and SSC regions. We also identified 45–60 SSRs, all of which are in the LSC and SSC regions (Appendix S5); 39–57 SSRs are mononucleotides, and 2–6 are dinucleotides. In addition, one SSR is tri‐nucleotide (*P. ferruginea*), and one is tetra‐nucleotide (*P. goudotiana* and *P. santosii*). We detected 4–10 compound SSRs in the *Puya* plastomes with a maximum interruption of 100 bp. The most abundant motif was poly‐A/T in a proportion of 86%–94%. The 16–17 large repeats were recorded in most *Puya* plastomes: 4–5 forward, 0–1 reverse, 11–13 palindromic, and 0–1 complement repeats (Appendix S6). Most dispersed repeats are distributed in the intergenic spacer regions of the genome, located mainly in the LSC region. The most variable region was the intergenic spacer *trnS* ^*GCU*^‐*trnG* ^*GCC*^ region, including forward, palindromic, and reverse complement repeats.

### 3.3 Genomic diversity and selection pressure of *Puya*

**FIGURE 5:**
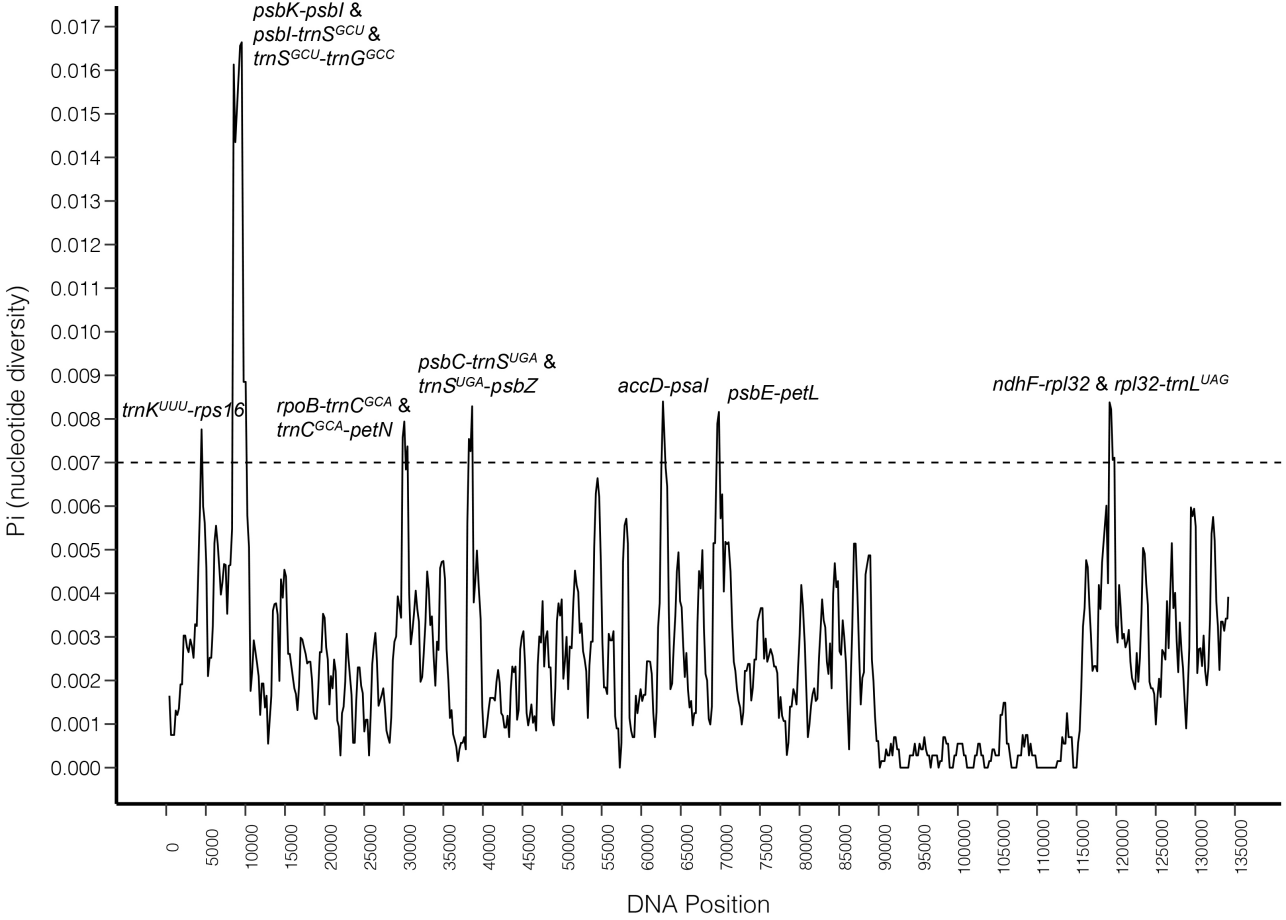
Nucleotide diversity of the entire plastome of 17 studied *Puya* accessions. *X*‐axis: Position with the unit of 5000 bp; *Y*‐axis: Nucleotide diversity of each window. Only the regions with the highest nucleotide diversities (*pi* > .007) were labeled.

The sliding window analyses using DnaSP software revealed highly variable regions in the plastome of *Puya* (Figure 5). When 17 plastomes of *Puya* were compared, the average value of nucleotide diversity over the entire plastome was 0.00244; the LSC (0.00287) and SSC (0.00342) regions exhibited higher diversity than the IR regions (0.00037). The most variable region is the intergenic spacer *psbK*‐*trnG* ^*GCC*^, including *psbK*‐*psbI*, *psbI*‐*trnS* ^*GCU*^, and *trnS* ^*GCU*^‐*trnG* ^*GCC*^. Additional highly variable regions include the following nine regions: *trnK* ^*UUU*^‐*rps16*, *rpoB*‐*trnC* ^*GCA*^, *trnC* ^*GCA*^‐*petN*, *psbC*‐*trnS* ^*UGA*^, *trnS* ^*UGA*^‐*psbZ*, *accD*‐*psaI*, *psbE*‐*petL*, *ndhF*‐*rpl32*, and *rpl32*‐*trnL* ^*UAG*^.

The ratio of dN/dS is a measure to detect selection. The value of dN/dS <1, =1, >1 indicates negative purifying selection, neutral evolution, and positive selection, respectively. The results showed that the dN/dS value in *Puya* is 0.0001 for 18 protein‐coding genes, 0.02–0.83 for 44 genes, 1.2–2.6 for four genes, and >25 for 12 genes (Appendix S7). However, since the sequences were so similar, all the dS values were less than 0.01 (Appendix S7), suggesting that our estimates of dN/dS may be unreliable (https://www.protocols.io/view/introduction‐to‐calculating‐dn‐ds‐ratios‐with‐code‐qhwdt7e?step=4). Since positive selection was unlikely to affect all sites of one gene over a prolonged time, we targeted our detection to some specific sites under positive selection using site models, which allowed the ratio of dN/dS to vary among codons. We found seven sites spanning four genes (*rbcL*, *rpoC1*, *rpl20*, and *ycf1*) exhibiting site‐specific selection (Table 3). Of these genes, the *rbcL* gene harbored two sites under positive selection, with three in *rpoC1*, two in *rpl20*, and one in *ycf1* (Table 3).

**TABLE 3:** Positive selection sites identified in the plastome of *Puya*. The log likelihoods (Ln L) and the *p*‐value of the likelihood ratio test (LRT) were calculated for every two models. The positive sites with * are significant (.001 < *p* < .05), those with ** are highly significant (*p* < .001)

Gene	Model	Ln L	Model compared	LRT *p*‐value	Positive sites
*rbcL*	M0	−2066.666391	M0 vs. M3	0.002840096	
	M3	−2058.597639			
	M1a	−2063.168252	M1a vs. M2a	0.010351747	
	M2a	−2058.597652			
	M7	−2063.522794	M7 vs.M8	0.011511624	328 S 0.989*,449 S 0.955*
	M8	−2059.058396			
			M8a vs.M8	0.004143805	
	M8a	−2063.168246			
*rpoC1*	M0	−2925.656370	M0 vs. M3	0.000016691	
	M3	−2911.968705			
	M1a	−2920.577409	M1a vs. M2a	0.000182510	
	M2a	−2911.968705			
	M7	−2921.340829	M7 vs.M8	0.000096104	85 K 0.996**,147 P 0.976*, 565 R 0.969*
	M8	−2912.090752			
			M8a vs.M8	0.000037936	
	M8a	−2920.576735			
*rpl20*	M0	−522.576368	M0 vs. M3	0.000001545	
	M3	−506.349106			
	M1a	−517.831161	M1a vs. M2a	0.000010314	
	M2a	−506.349106			
	M7	−524.203311	M7 vs.M8	0.000000018	73 K 0.954*,76 N 1.000**
	M8	−506.370000			
			M8a vs.M8	0.000003541	
	M8a	−517.119215			
*ycf1*	M0	−8279.028189	M0 vs. M3	0.000004952	
	M3	−8264.040700			
	M1a	−8271.139669	M1a vs. M2a	0.000883916	
	M2a	−8264.108520			
	M7	−8271.364612	M7 vs.M8	0.007586158	545 L 0.997**
	M8	−8266.483182			
			M8a vs.M8	0.002273789	
	M8a	−8271.140305			

### 3.4 Phylogenetic analyses

**FIGURE 6:**
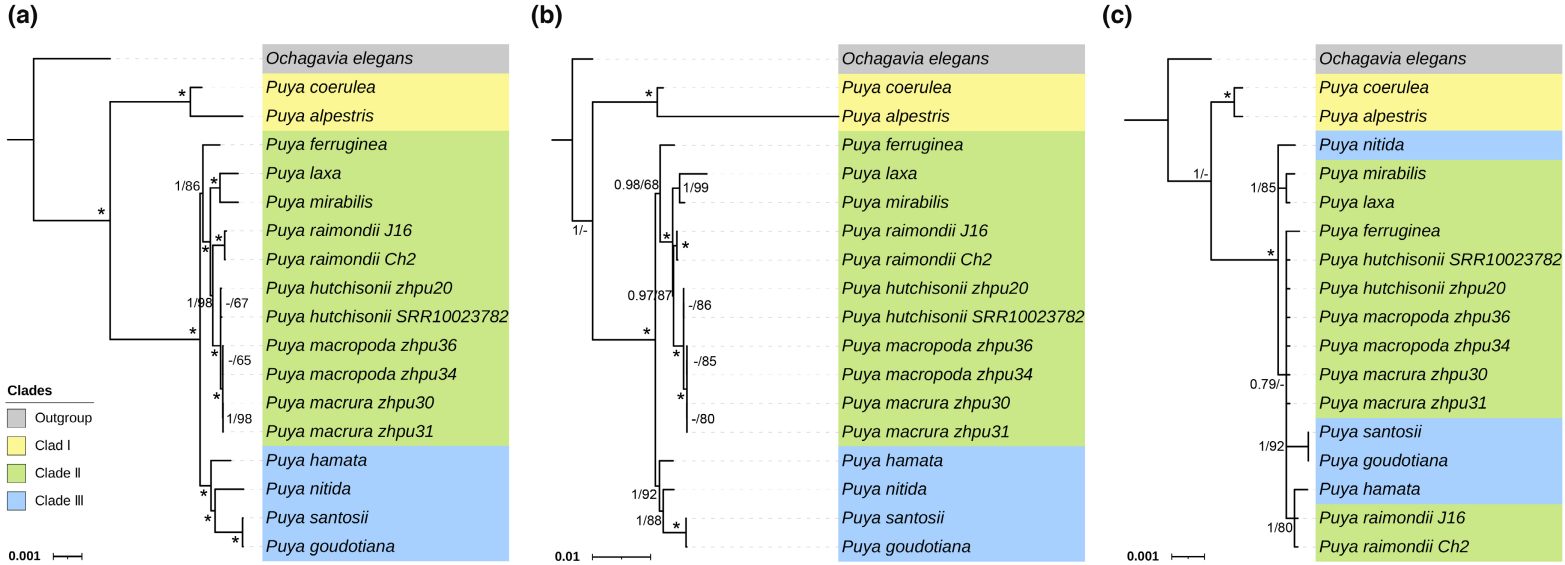
Phylogenetic relationships of 17 *Puya* accession inferred from three types of datasets. (a) Complete plastomes (including LSC, SSC, and IRb regions). (b) A combine of 12 hypervariable regions (*psbK*‐*psbI*, *psbI*‐*trnS* ^*GCU*^, *trnS* ^*GCU*^‐*trnG* ^*GCC*^, *trnK* ^*UUU*^‐*rps16*, *rpoB*‐*trnC* ^*GCA*^, *trnC* ^*GCA*^‐*petN*, *psbC*‐*trnS* ^*UGA*^, *trnS* ^*UGA*^‐*psbZ*, *accD*‐*psaI*, *psbE*‐*petL*, *ndhF*‐*rpl32*, and *rpl32*‐*trnL* ^*UAG*^). (c) Three standard barcodes (*rbcL*, *matK*, and *trnH*‐*psbA*). *Ochagavia elegans* is outgroup. Numbers at nodes indicate posterior probabilities (PP, left) and bootstrap support values (BS, right), separately. Branches with * have PP = 1 and BS = 100% and with — have a low support rate (values <0.6/60% not shown). The colors represent different clades.

The BI and ML analyses revealed the phylogenetic relationships among *Puya* species (Figure 6). The topology of the BI tree is almost congruent with the ML tree in each of the three datasets. Based on the complete plastomes dataset, the tree is consistent with the tree generated from the hypervariable regions but differs from that based on the standard barcodes. The highest support values (posterior probabilities/PP = 1, bootstrap support/BS ≥98% mostly) were obtained when using the complete plastome, with slightly weaker support in the hypervariable regions (PP >0.97, BS ≥87% mostly), and most relationships were not fully resolved in the standard barcodes. Therefore, the relationships among species described below are based on the complete plastome dataset.

Interspecific relationships among the species examined were strongly supported for the most part (PP = 1, BS ≥98%, Figure 6a), and conspecific samples from *P. hutchisonii*, *P. macropoda*, *P. macrura*, and *P. raimondii*, clustered together, respectively. The 17 *Puya* accessions clustered into three well‐supported clades: Clade I (PP = 1, BS = 100%) is at the base of the genus, including *P. alpestris* and *P. coerulea*, and is sister to Clade II + Clade III; Clade II (PP = 1, BS = 86%) consists of *P. ferruginea*, *P. hutchisonii*, *P. laxa*, *P. macropoda*, *P. macrura*, *P. mirabilis*, and *P. raimondii*; Clade III (PP = 1, BS = 100%) consists of *P. goudotiana*, *P. hamata*, *P. nitida*, and *P. santosii*. The genus *Puya* is monophyletic with strong support (PP = 1, BS = 100%), whereas the two subgenera, *Puya* and *Puyopsis*, are nonmonophyletic.

### 3.5 Analyses of potential barcodes

We evaluated the efficacy of the three datasets (the complete plastomes, the combination of 12 hypervariable regions, and three standard barcodes) to discern *Puya* species. Based on the phylogenetic trees, species identification was considered successful (100% of the species resolved) only if all conspecific individuals formed a monophyletic clade with more than 50% BS support. For the hypervariable dataset (BS ≥80%) and the complete plastomes dataset (BS ≥65%), the four species with two accessions (i.e., *P. hutchisonii*, *P. macropoda*, *P. macrura*, and *P. raimondii*) were correctly identified to species (Figure 6a,b). However, the tree based on the standard barcodes dataset (*rbcL* + *matK* + *trnH*‐*psbA*) failed to distinguish all these species (Figure 6c). Thus, we concluded that the hypervariable regions and the plastomes of *Puya* were potential barcodes for assigning species in *Puya*. If the hypervariable regions provide insufficient resolution in some circumstances, plastomes may provide more characters. However, the three standard barcodes showed to be poor candidates as DNA barcodes for *Puya*.

### 3.6 Divergence time estimation

**FIGURE 7:**
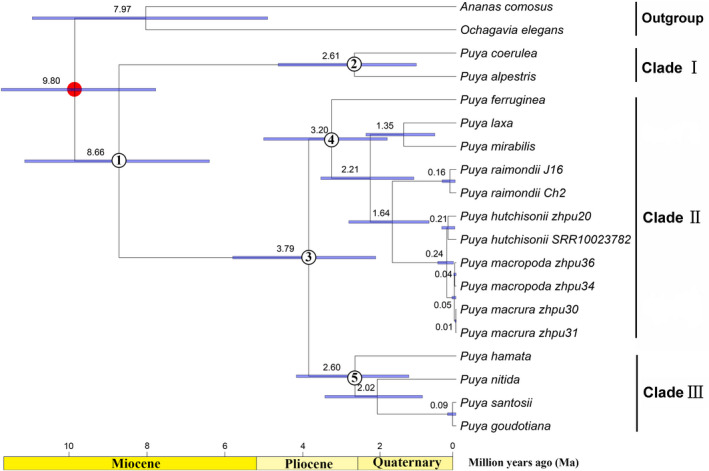
*Puya* time‐tree estimated using BEAST2. Numbers next to nodes indicate the median age. Blue bars represent the 95% highest posterior density (HPD) intervals of node ages. The 95% HPD intervals for estimated age of node 1–5 are: Node 1 = 6.34–11.08 Ma, node 2 = 1.02–4.57 Ma, node 3 = 2.07–5.74 Ma, node 4 = 1.77–4.94 Ma, node 5 = 1.21–4.10 Ma, and root node = 7.72–11.68 Ma. One secondary time‐calibration point (Givnish et al., 2011) was marked by red dots in the tree.

Based on the complete plastomes dataset, the divergence time was estimated for the 13 species of *Puya*, representing the significant clades (Figure 7). Ancestral *Puya* diverged from Bromelioideae in the Miocene, at approximately 9.80 Ma (7.72–11.68 Ma, 95% highest posterior density). The estimated crown age of extant *Puya* was 8.66 Ma (6.34–11.08 Ma) in the late Miocene. Within *Puya*, the crown ages of Clade I, Clade II, and Clade III were estimated in the Pliocene‐Pleistocene, at 2.61 Ma (1.02–4.57 Ma), 3.20 Ma (1.77–4.94 Ma), and 2.60 Ma (1.21–4.10 Ma), respectively. Furthermore, the crown age of Clade II + Clade III was dated as 3.79 Ma (2.07–5.74 Ma).

## 4 DISCUSSION

### 4.1 Plastome features

The 12 newly generated *Puya* plastomes were highly conserved and ranged from 159,542 to 159,839 bp. They all exhibited the typical plastome structure, gene order, and content (Figure 2). Compared to plastomes of other genera in the Bromeliaceae, *Puya* plastomes were smaller than *Fascicularia* (161,423 bp; Paule et al., 2020) and similar to *Ananas* (159,636 bp; Nashima et al., 2015; Redwan et al., 2015) and *Tillandsia* (159,659 bp; Poczai & Hyvonen, 2017) in size. The GC content of *Puya* (37.3%–37.4%) was similar to that of *Fascicularia* (37.3%; Paule et al., 2020), *Tillandsia* (37.2%; Poczai & Hyvonen, 2017), and *Ananas* (37.4%; Nashima et al., 2015; Redwan et al., 2015). The number of SSRs and dispersed repeats in the *Puya* plastome (45–60, 8–22, Appendix S5 and S6) was fewer than in *A. comosus* (205, 35) (Redwan et al., 2015).

Generally, plastomes in land plants have conserved quadripartite structures, including LSC, SSC, and a pair of IR regions. The contraction and expansion of the IR regions contribute to the variation in plastome size, driving plant diversity (Luo et al., 2016). The length of the IR regions varied from 26,738 to 26,779 bp (Table 1) in *Puya* plastomes. In comparison to other Bromeliads, the plastome sequence of *Puya* did not show any significant expansion or contraction of the IR regions (Figure 3). At the SSC/IRb region, the *Puya* species share synapomorphic structural features with other Bromeliads, including a truncated *ycf*1 gene (with 1089 bp located at IRb; Poczai & Hyvonen, 2017; Redwan et al., 2015). From the stability of IR regions in *Puya* and other Bromeliads, it could be inferred that they have not suffered noticeable expansions or contractions.

### 4.2 Plastome evolution in *Puya*

*Puya* is characterized by rapid diversifications, leading to the challenge of reconstructing its evolutionary history (Givnish et al., 2011). Among 17 *Puya* accessions, the nucleotide identity of all CDSs is nearly 99%, showing few differences in this genus. Thus, the hypervariable regions with relatively high genetic variation may serve as effective markers for elucidating phylogenetic relationships and species discrimination in *Puya*. In previous studies, the intergenic spacer of the *rpoB* and *psbD* gene regions show high divergence in pineapple (Redwan et al., 2015). Compared to the pineapple plastome, two regions of *rpoB‐psbD* in *Puya* showed high nucleotide diversity, including *rpoB‐trnC* ^*GCA*^ and *trnC* ^*GCA*^‐*petN*. We detected 10 additionally highly variable regions, including *trnK* ^*UUU*^‐*rps16*, *psbK*‐*psbI*, *psbI*‐*trnS* ^*GCU*^, *trnS* ^*GCU*^‐*trnG* ^*GCC*^, *psbC*‐*trnS* ^*UGA*^, *trnS* ^*UGA*^‐*psbZ*, *accD*‐*psaI*, *psbE*‐*petL*, *ndhF*‐*rpl32*, and *rpl32*‐*trnL* ^*UAG*^. Collectively, we suggest these 12 hypervariable sequences may act as useful markers for phylogenetic studies in *Puya*.

Plastid genes are essential to plant metabolism, and genes under negative selection tend to maintain protein functions, while those under positive selection may drive adaptive divergence (Burri et al., 2010; Piot et al., 2018). Although most plastomes are highly conserved in sequence and structure within angiosperms, with dN/dS values less than 1 (Jansen et al., 2007; Wicke et al., 2011), our results showed that four protein‐coding genes (*rbcL*, *rpoC1*, *rpl20*, and *ycf1*) undergo significant site‐specific selection pressure (Table 3). Recent studies indicate that positive selection in these four genes may be a widespread phenomenon; *rbcL* and *rpl20* in Bromeliaceae (Hermida‐Carrera et al., 2020; Redwan et al., 2015), *rpoC1* in Zingiberaceae (Li, Li, et al., 2021), and *ycf1* in Asteraceae (Kim et al., 2020).

While the *ycf1* gene is required for photosynthetic protein import and essential for plant fitness and viability (de Vries et al., 2015), the large subunit of Rubisco encoded by the *rbcL* gene is the cornerstone of photosynthesis and is responsible for converting inorganic carbon into organic compounds (Spreitzer & Salvucci, 2002). Here, positive selection in the *rbcL* gene of terrestrial land plants is ubiquitous (Kapralov & Filatov, 2007), reflecting adaptation to environmental changes and the corresponding co‐evolution of proteins in the Rubisco complex (Tcherkez et al., 2006). In addition, the *rpoC* gene encodes the major catalytic subunit of RNA polymerase, which is essential for plastome biogenesis (Liebers et al., 2018), and the *rpl20* gene encodes large ribosomal subunits, which is indispensable in ribosome biogenesis, protein synthesis, cell growth, development, and apoptosis (Gao et al., 2019; Saha et al., 2017). Our finding that all four of these genes are under positive selection may indicate that natural selection targets different plastid functions, supporting the possible involvement of plastid genes in adaptation and speciation processes (Greiner & Bock, 2013).

### 4.3 Phylogenomics application

Plastome data are widely used to construct plant phylogenies (Davis et al., 2014; Li et al., 2019) mainly because they can resolve phylogenetic relationships among recent and rapid diverging groups (Zhao et al., 2021). Based on molecular markers, including nuclear and plastid sequences and AFLP data (Givnish et al., 2011; Jabaily & Sytsma, 2010, 2013), only two significant clades, “Core *Puya*” and “Chilean *Puya*,” were recovered with robust support. In this study, three well‐supported clades were clustered geographically (Figure 6a), with Clade I distributed in Chile, Clade II ranging from Bolivia to Ecuador, and Clade III in Colombia (except for *P. hamata* which ranges from Colombia to Peru) corresponding to “Chilean *Puya*,” “Central Andes *Puya*,”, and “Northern Andes *Puya*” (Jabaily & Sytsma, 2010). This strong spatial signature may indicate that the *Puya* species in our study might have experienced two geographically delimited and mostly disconnected radiations in Chile and the high Andes, respectively, followed by dispersal events to the Northern Andes. Similar patterns have been found in other Andes species, for example, Espeletiinae (Pouchon et al., 2018). Given the limited sampling regime in our study, we acknowledge that more *Puya* species need to be collected to verify that the geographic patterns found here also hold for all 229 species in the genus.

Both interspecific and intraspecific relationships among the studied *Puya* species were well resolved. Our results also corroborated previous studies that the genus *Puya* is monophyletic, and the two subgenera are nonmonophyletic (Jabaily & Sytsma, 2010), as subg. *Puya* species *P. alpestris* in Clade I and *P. raimondii* in Clade II are not as sister species. These results highlight the possible independent evolution of the sterile inflorescence apex in subg. *Puya* species. It has been previously suggested that the sterile inflorescence apex may act as feeding stations and perches for passerine and hummingbird pollinators (Hornung‐Leoni et al., 2013). Thus, this character may not be an appropriate diagnostic of subg. *Puya* since it seems to have experienced several convergent evolutions events to attract pollinators (Anderson et al., 2005; Hornung‐Leoni & Sosa, 2008; Jabaily & Sytsma, 2010; Johow, 1898). A similar trend has been proposed recently for the evolution of the Andean genus *Espeletia* (Pouchon et al., 2018).

In mesangiospermae, conflicting topologies between the plastome and nuclear trees were widely detected due to their inconsistent genetic patterns (Leebens‐Mack et al., 2019; Li et al., 2019; Ma et al., 2021; Yang et al., 2020). Several processes could explain ambiguous and conflicting topologies, such as hybridization, polyploidization, and incomplete lineage sorting (ILS; Degnan & Rosenberg, 2009; Paule et al., 2020; Rieseberg & Soltis, 1991; Ye et al., 2021). Comparing our plastid trees with biparentally inherited nuclear gene trees (Liu et al., 2021), incongruences were observed in the placement of *P. raimondii* and *P. macrura*. In the plastome phylogeny, *P. raimondii* is sister to the clade comprising *P. hutchisonii*, *P. macropoda*, and *P. macrura*, and *P. macrura* is sister to *P. macropoda*. However, a different topology built with nuclear genes weakly rejected the sister relationships between these species (Liu et al., 2021). The sparse sampling has greatly limited our understanding of phylogenetic discordances in this study. ILS is a random process that should not necessarily lead to the geographic footprint (Xu et al., 2021) in the phylogenetic clustering of plastid markers that we demonstrated here. ILS is not likely to explain the cytonuclear discordance observed in *Puya*, as is the case for high‐elevation Andes *Espeletia* (Pouchon et al., 2018). Polyploidy is not known in *Puya* and is very rare in Bromeliaceae (Brown & Gilmartin, 1989; Gitaí et al., 2005; Smith & Downs, 1974), suggesting that it does not account for the discordance. Nevertheless, there is a reasonable doubt that polyploidy has not yet been revealed in *Puya*, offering an incentive for future explorations.

Hybridization is common in plants and has a principal role in the origin of new species (Mallet, 2007; Xu et al., 2021). In the high‐elevation Andes, hybridization is also a relatively common occurrence, rendered by “flickering connectivity” and the diversity of habitats, resulting from Pleistocene climate changes and the steep ecological clines along mountain slopes, respectively (Schley et al., 2021). The hybridization hypothesis stands out as the most probable explanation for the biogeographic patterns and the extremely short branch length observed between *P. macrura* and *P. macropoda* (Figure 6a). *Puya* is characterized by bird pollination and winged seeds, providing extensive pollen and seed dispersal and a strong connection among populations (Smith & Downs, 1974). Given that recent hybridization among other *Puya* species has been verified (Jabaily & Sytsma, 2010, 2013), assessing the magnitude of gene flow or hybridization in further phylogenetic studies of *Puya*, or even in the family Bromeliaceae (Matallana et al., 2016; Wendt et al., 2008), is warranted.

### 4.4 Screening of potential DNA barcodes

DNA barcoding is a valuable tool for species identification (Hebert et al., 2003). Four plant DNA barcode markers, *rbcL*, *matK*, *trnH*‐*psbA*, and ITS2, have been suggested as the plant standard barcodes (Kress, 2017). However, for DNA barcoding, discriminating among taxa with complex recent radiations remains a difficult challenge (Kress, 2017; Spooner, 2009). Our study showed that the combination of *rbcL*, *matK*, and *trnH*‐*psbA* sequences has relatively low genetic variability and low species resolution in the genus *Puya* (Figure 6c). Because of the inherent limitation of standard barcodes and recent decreases in sequencing costs, complete plastome data is a useful tool for the next generation of DNA barcodes that have higher interspecific and lower intraspecific divergence (Barrett et al., 2016; Coissac et al., 2016; Huang et al., 2014; Ji et al., 2019; Knox, 2014; Li et al., 2015; Moore et al., 2010; Ruhsam et al., 2015; Song et al., 2020; Zhang et al., 2021). We found more variable characters in the complete plastomes of *Puya*, and most interspecific relationships were resolved with robust support (Figures 5 and 6a); four species, with two accessions each, were correctly identified using their complete plastomes. Therefore, we suggest using complete plastomes as a practical “super‐barcode” for phylogenetic reconstruction and species identification in the genus *Puya*.

Taxon‐specific barcodes may also enhance species discrimination rates because they typically provide more genetic information within a particular group of species than standard DNA barcodes used across taxa of broad phylogenetic dispersion (Dong et al., 2021). Considering this factor, taxon‐specific barcodes with sufficiently high mutation rates are now widely used, representing an intermediate trade‐off between standard barcode DNA and “super‐barcodes” (Li et al., 2015; Li, Gichira, et al., 2021). Our results also show that the resolution of hypervariable regions was similar to that of the complete plastomes (Figure 6a,b), which indicates that the 12 hypervariable regions screened in this study can also be used as DNA barcodes for phylogenetic applications and phylogeographic investigations.

### 4.5 Divergence of *Puya*

Unsurprisingly, the divergence time estimates (stem and crown age of *Puya*) under a secondary calibrated crown node agreed with previous studies (Givnish et al., 2011; Möbus et al., 2021). Our results indicate that *Puya* segregated from Bromelioideae (ca. 9.80 Ma) and began to diversify (ca. 8.66 Ma) in the late Miocene (Figure 7), followed by diversification from the middle Pliocene to the Pleistocene. Like many other plant groups in the high Andes, for example, *Hedyosmum* (Antonelli & Sanmartin, 2011), *Lupinus* (Drummond et al., 2012; Nevado et al., 2018), *Espeletia* (Pouchon et al., 2018, 2021), Lobelioideae (Lagomarsino et al., 2016), and *Phlegmariurus* (Testo et al., 2019), the orogeny of the Andes and climate change might have contributed to the diversification of *Puya*. Geological evidence indicates that rapid uplift of the Central Andes occurred during the last 10 Myr (million years), which essentially reshaped the ecological system and produced complex high elevation habitats (Garzione et al., 2006, 2008, 2014; Graham, 2010; Gregory‐Wodzicki, 2000; Hughes, 2016). Such mountain building promoted the formation of a barrier for Amazonian moisture, intensifying aridity and seasonality on the western slopes (Houston & Hartley, 2003). Thus, the final and recent rise of the Andes since the late Miocene may have driven the formation of suitable habitats for *Puya* species and segregated the Andean *Puya* from the Chileans (Jabaily & Sytsma, 2010).

The Pleistocene glacial cycles have also been suggested to promote population expansion and contraction, driving a high speciation rate, especially in mountainous areas (Kadereit & Abbott, 2021). In the high Andes, glacial cycles were primarily associated with vertical and minimal horizontal displacement of vegetation zones, causing a downward shift of 1200–1400 m in the treeline at the Last Glacial Maximum and an upward shift in elevation during interglacial periods (Graham, 2009; Hooghiemstra et al., 2006; Jomelli et al., 2014; Nevado et al., 2018; Simpson, 1974; Van der Hammen & Cleef, 1986). The occurrence of close relatives of *Puya* at different elevations at the same latitude and frequent transitions between adjacent cordilleras lends support to the proposal that Pleistocene glaciation cycles were responsible for allopatric speciation in this group via a glacial “pump” (Jabaily & Sytsma, 2013). Therefore, we agree with Jabaily and Sytsma (2010, 2013) that the recent uplift of the Andes since the late Miocene and subsequent Pleistocene glaciation cycles may have triggered the origin of the major clades delimited in this study (i.e., Clade I, Clade II, and Clade III) and recently rapid speciation in *Puya*.

### 4.6 Limitations of this study

We are aware that our sampling regime is limited to 13 of the 229 species of *Puya*, and the three main clades found in this study, corresponding to “Chilean *Puya*,” “Central Andes *Puya*,” and “Northern Andes *Puya*,” may not represent the full diversity of taxa in the genus *Puya*. Thus, the geographic patterns found in our study should be further investigated by an increased sampling, especially for those species distributed on the border of Chile. Moreover, although orogeny and climate change are often considered to be the major driving factors for the diversification of species in the high Andes (Hoorn et al., 2022), and the estimated divergence time in this study is consistent with these occurrences, other biotic factors (e.g., pollinator interactions and competition), likely contribute to the patterns found in this study. Future studies should include quantitative ecological approaches that will help discern additional potential sources of variation driving the diversification of *Puya* in the Andes.

## 5 CONCLUSIONS

We reported 12 newly generated plastomes and compared them with five published ones, representing 13 *Puya* species. Comparative analyses of *Puya* plastomes, representing the major clades, suggested that the quadripartite structure and identical gene content were conserved. The genes of *rbcL*, *rpoC1*, *rpl20*, and *ycf1* might be related to habitat adaptation and plant growth and reproduction under selection pressure. We tested complete plastomes, hypervariable regions, and standard DNA barcodes for phylogenetic recontraction and species discrimination. The phylogenetic tree built by the former two datasets provided stronger discrimination power and support for three major clades: “Chilean *Puya*,” “Central Andes *Puya*,” and “Northern Andes *Puya*.” Twelve hypervariable regions could be used as potential DNA barcodes for the genus *Puya*. Among these datasets, the complete plastomes greatly improved species resolution in *Puya* and showed high potential for future studies. Divergence time within *Puya* shed insight on the rapid radiation of this genus related to Andean orogeny and Pleistocene climate oscillations.

## AUTHOR CONTRIBUTIONS

**Lu Liu:** Data curation (lead); formal analysis (lead); writing – original draft (lead); writing – review and editing (equal). **Yu‐Qu Zhang:** Investigation (equal); writing – review and editing (equal). **Liscely Tumi:** Investigation (equal); writing – review and editing (equal). **Mery Luz Suni:** Writing – review and editing (equal). **Mónica Arakaki:** Writing – review and editing (equal). **Kevin Burgess:** Writing – review and editing (equal). **Xuejun Ge:** Funding acquisition (lead); investigation (equal); project administration (lead); writing – original draft (equal); writing – review and editing (lead).

## CONFLICT OF INTEREST

The authors declare that they have no competing interests.

## Supporting information


Appendix S1
Appendix S2
Appendix S3
Appendix S4
Appendix S5
Appendix S6
Appendix S7

## Data Availability

Twelve assembled plastomes and their raw sequencing data described in this article are publicly available in the National Center for Biotechnology Information (NCBI) database under project PRJNA784015 at https://www.ncbi.nlm.nih.gov/bioproject/PRJNA784015. All plastomes are also released in the Science Data Bank at http://www.doi.org/10.11922/sciencedb.01357. GenBank and SRA accession numbers are provided in Table 1.
