# Supplementary material for: Genetic markers in Andean Puya species (Bromeliaceae) with implications on plastome evolution and phylogeny
Source: Ecol Evol. 2022 Jul 29;12(8):e9159. doi: 10.1002/ece3.9159 (PMC9336176; doi:10.1002/ece3.9159)
Supplement: Supplementary file 1 — Appendix S1 Appendix S2 Appendix S3 Appendix S4 Appendix S5 Appendix S6 Appendix S7 [file ECE3-12-e9159-s001.docx]

Appendix S1 The diagnose of studied *Puya* species. The blank cells with “—” indicate missing data.

| Species | Leaf length (cm) | Life form | Height (cm) | Inflorescence length (cm) | Flower color | Leaf indument | Flowers at the apex | Reference |
| --- | --- | --- | --- | --- | --- | --- | --- | --- |
| *P. alpestris* | 36-120 | terrestrial | 120-500 | 25-100 | blue to metallic blue-green | densely lepidote beneath or glabrous above | sterile | Gómez Romero and Alfredo Grau,  http://www.scielo.org.ar/scielo.php?script=sci_arttext&pid=S1851-23722009000100013；  Jabaily and Sytsma, doi: 10.3732/ajb.0900107;  Zizka et al., doi: 10.1007/s12228-012-9290-9 |
| *P. coerulea* | 11-62 | lithophyte | 100-270 | 50-110 | blue to blue-violet | scales | fertile | Hornung-Leoni et al., doi: 10.1016/j.actao.2013.05.010;  Jabaily and Sytsma, doi: 10.3732/ajb.0900107;  Zizka et al., doi: 10.1007/s12228-012-9290-9 |
| *P. ferruginea* | 100 | lithophyte | 250 | — | greenish white to various shades of purple | white-lepidote beneath | fertile | Ayasta, et al., doi: 10.15381/rpb.v28i2.18115 |
| *P. goudotiana* | 140-170 | — | 500 | 100-200 | greenish blue | glabrous | fertile | Madriñán, doi: 10.18257/raccefyn.223 |
| *P. hamata* | 70-150 | lithophyte | 400 | 400 | dark blue | minutely lepidote beneath | fertile | Ayasta et al., doi: 10.15381/rpb.v28i2.18115;  Garcia-Meneses and Ramsay, doi: 10.15446/caldasia.v36n1.43891;  Smith, https://www.biodiversitylibrary.org/page/397671#page/1/mode/1up |
| *P. hutchisonii* | >60 | — | 150 | 40-50 | metallic-blue | scales on both sides | fertile | Smith, https://www.biodiversitylibrary.org/page/14502208#page/147/mode/1up |
| *P. laxa* | 27 | stony soil | 80 | — | dark violet | densely scale beneath | fertile | Smith, https://www.biodiversitylibrary.org/page/12996319#page/209/mode/1up |
| *P. macropoda* | — | lithophyte | 200 | 100 | yellow-green | finely white-stellate, glabrous with age | fertile | Smith, https://www.biodiversitylibrary.org/page/14502208#page/148/mode/1up |
| *P. macrura* | 40 | lithophyte | 100-150 | 35 | dark violet | scales on both sides | fertile | Carl Mez, https://www.biodiversitylibrary.org/page/233255#page/23/mode/1up |
| *P. mirabilis* | 22-65 | lithophyte | 50-200 | 8-50 | white-cream or green-yellow | glabrous | fertile | Gómez Romero and Alfredo Grau,  http://www.scielo.org.ar/scielo.php?script=sci_arttext&pid=S1851-23722009000100013 |
| *P. nitida* | 35-38 | terrestrial | 200 | 58-62 | light green | glabrous | fertile | Hornung-Leoni and Sosa, doi: 10.1007/s00606-005-0302-z;  Madriñán, doi: 10.18257/raccefyn.223 |
| *P. raimondii* | 123-125 | lithophyte and paramo | 950-1200 | 430-500 | creamy | white scales | sterile | Hornung-Leoni et al., doi: 10.1016/j.actao.2013.05.010 |
| *P. santosii* | 31-37 | — | 200 | 32-50 | greenish blue | glabrous | fertile | Madriñán, doi: 10.18257/raccefyn.223 |

Appendix S2 Molecular models selected for three datasets. Twelve hypervariable regions include *psbK*-*psbI*, *psbI*-*trnS^GCU^*, *trnS^GCU^*-*trnG^GCC^*, *trnK^UUU^*-*rps16*, *rpoB*-*trnC^GCA^*, *trnC^GCA^*-*petN*, *psbC*-*trnS^UGA^*, *trnS^UGA^*-*psbZ*, *accD*-*psaI*, *psbE*-*petL*, *ndhF*-*rpl32*, and *rpl32*-*trnL^UAG^*.

| Dataset | Best fit model | Model selection |
| --- | --- | --- |
| Plastome | TVM+I+G | GTR+G+I |
| *rbcL* + *matK* + *trnH-psbA* | GTR+G | GTR+G |
| Twelve hyper-variable regions | GTR+G | GTR+G |

Appendix S3 Relative synonymous codon usage (RSCU) of 17 *Puya* accessions. Amino acid abbreviations follow the genetic code (http://www.biology-pages.info/C/Codons.html).

| Codon | Amino acid | Count | RSCU | Codon | Amino acid | Count | RSCU |
| --- | --- | --- | --- | --- | --- | --- | --- |
| GCA | Ala | 392-398 | 1.132-1.148 | CCA | Pro | 321-325 | 1.164-1.176 |
| GCC | Ala | 203-208 | 0.588-0.6 | CCC | Pro | 225-228 | 0.816-0.824 |
| GCG | Ala | 147-150 | 0.428-0.436 | CCG | Pro | 133-134 | 0.48-0.484 |
| GCT | Ala | 634-637 | 1.828-1.844 | CCT | Pro | 420-422 | 1.52-1.532 |
| TGC | Cys | 80-82 | 0.492-0.504 | CAA | Gln | 703-709 | 1.492-1.502 |
| TGT | Cys | 243-245 | 1.496-1.508 | CAG | Gln | 234-241 | 0.498-0.508 |
| GAC | Asp | 210-213 | 0.378-0.384 | AGA | Arg | 526-536 | 1.908-1.938 |
| GAT | Asp | 899-908 | 1.616-1.622 | AGG | Arg | 162-168 | 0.588-0.606 |
| GAA | Glu | 1040-1043 | 1.448-1.454 | CGA | Arg | 368-376 | 1.332-1.362 |
| GAG | Glu | 392-398 | 0.546-0.552 | CGC | Arg | 93-99 | 0.336-0.36 |
| TTC | Phe | 588-591 | 0.78-0.788 | CGG | Arg | 119-127 | 0.432-0.462 |
| TTT | Phe | 907-918 | 1.212-1.22 | CGT | Arg | 367-371 | 1.332-1.344 |
| GGA | Gly | 748-751 | 1.66-1.668 | AGC | Ser | 102 | 0.288-0.294 |
| GGC | Gly | 153-155 | 0.34-0.344 | AGT | Ser | 414-416 | 1.176-1.188 |
| GGG | Gly | 290-294 | 0.644-0.652 | TCA | Ser | 443-448 | 1.266-1.278 |
| GGT | Gly | 605-609 | 1.344-1.348 | TCC | Ser | 357-364 | 1.014-1.038 |
| CAC | HIS | 149-151 | 0.452-0.46 | TCG | Ser | 198-202 | 0.564-0.576 |
| CAT | HIS | 505-510 | 1.54-1.548 | TCT | Ser | 580-586 | 1.656-1.668 |
| ATA | Ile | 706-710 | 0.915-0.921 | ACA | Thr | 442-446 | 1.264-1.276 |
| ATC | Ile | 489-497 | 0.636-0.645 | ACC | Thr | 257-262 | 0.736-0.752 |
| ATT | Ile | 1109-1114 | 1.44-1.443 | ACG | Thr | 146-153 | 0.42-0.44 |
| AAA | Lys | 1022-1024 | 1.45-1.458 | ACT | Thr | 541-545 | 1.552-1.56 |
| AAG | Lys | 381-389 | 0.542-0.55 | GTA | Val | 553-557 | 1.512-1.52 |
| CTA | Leu | 391-397 | 0.852-0.864 | GTC | Val | 175-179 | 0.48-0.488 |
| CTC | Leu | 195-202 | 0.426-0.438 | GTG | Val | 204-207 | 0.556-0.564 |
| CTG | Leu | 163-167 | 0.354-0.366 | GTT | Val | 524-529 | 1.432-1.444 |
| CTT | Leu | 582-588 | 1.266-1.284 | TGG | Trp | 459-463 | 1 |
| TTA | Leu | 830-834 | 1.806-1.818 | TAC | Tyr | 209-215 | 0.41-0.422 |
| TTG | Leu | 580-586 | 1.26-1.278 | TAT | Tyr | 803-812 | 1.578-1.59 |
| ATG | Met | 639-645 | 1 | TAA | Stop | 42-44 | 1.449-1.518 |
| AAC | Asn | 285-288 | 0.448-0.452 | TAG | Stop | 26-28 | 0.897-0.966 |
| AAT | Asn | 984-991 | 1.548-1.552 | TGA | Stop | 17 | 0.585 |

Appendix S4 Total number of tandem repeats identified within the plastomes of 17 *Puya* accessions

| Species | GenBank/SRA accession number | Period size (bp) | | | | | Count |
| --- | --- | --- | --- | --- | --- | --- | --- |
|  |  | <10 | 10-19 | 20-29 | 30-39 | ≥40 |  |
| *P. alpestris* | SRR13700326 | 1 | 43 | 25 |  |  | 69 |
| *P. coerulea* | SRR9846915 | 5 | 47 | 24 | 1 | 1 | 78 |
| *P. ferruginea* | OL639024 | 5 | 36 | 28 | 2 | 1 | 72 |
| *P. goudotiana* | OL639023 | 4 | 37 | 26 | 3 | 1 | 71 |
| *P. hamata* | MZ403751 | 2 | 37 | 27 | 3 | 1 | 70 |
| *P. hutchisonii* | OL639026 | 4 | 41 | 29 | 3 |  | 77 |
| *P. hutchisonii* | SRR10023782 | 4 | 40 | 30 | 3 |  | 77 |
| *P. laxa* | SRR13700325 | 1 | 39 | 26 | 3 |  | 69 |
| *P. macropoda* | OL639027 | 4 | 41 | 29 | 3 |  | 77 |
| *P. macropoda* | OL639028 | 4 | 41 | 29 | 3 |  | 77 |
| *P. macrura* | OL639025 | 4 | 41 | 29 | 3 |  | 77 |
| *P. macrura* | OL639019 | 4 | 41 | 29 | 3 |  | 77 |
| *P. mirabilis* | NC_045380.1 | 5 | 47 | 24 | 1 | 1 | 78 |
| *P. nitida* | OL639018 | 4 | 37 | 29 | 3 |  | 73 |
| *P. raimondii* | OL639020 | 3 | 39 | 29 | 3 | 1 | 75 |
| *P. raimondii* | OL639021 | 3 | 39 | 29 | 3 | 1 | 75 |
| *P. santosii* | OL639022 | 4 | 37 | 26 | 3 | 1 | 71 |

Appendix S5 Total number of simple sequence repeats (SSRs) identified within the plastomes of 17 *Puya* accessions

| Species | GenBank/SRA accession number | No. of SSRs | | | | | Count | Compound SSR |
| --- | --- | --- | --- | --- | --- | --- | --- | --- |
|  |  | A/T | C/G | AT/AT | AAT/ATT | AATAT/ATATT |  |  |
| *P. alpestris* | SRR13700326 | 42 | 1 | 6 |  |  | 49 | 7 |
| *P. coerulea* | SRR9846915 | 39 |  | 6 |  |  | 45 | 4 |
| *P. ferruginea* | OL639024 | 49 | 1 | 2 | 1 |  | 53 | 6 |
| *P. goudotiana* | OL639023 | 44 | 1 | 3 |  | 1 | 49 | 7 |
| *P. hamata* | MZ403751 | 44 | 1 | 2 |  |  | 47 | 5 |
| *P. hutchisonii* | OL639026 | 54 | 1 | 3 |  |  | 58 | 7 |
| *P. hutchisonii* | SRR10023782 | 54 | 1 | 3 |  |  | 58 | 8 |
| *P. laxa* | SRR13700325 | 50 | 1 | 3 |  |  | 54 | 10 |
| *P. macropoda* | OL639027 | 56 | 1 | 3 |  |  | 60 | 7 |
| *P. macropoda* | OL639028 | 56 | 1 | 3 |  |  | 60 | 8 |
| *P. macrura* | OL639025 | 56 | 1 | 3 |  |  | 60 | 8 |
| *P. macrura* | OL639019 | 56 | 1 | 3 |  |  | 60 | 8 |
| *P. mirabilis* | NC_045380.1 | 48 | 1 | 4 |  |  | 53 | 10 |
| *P. nitida* | OL639018 | 49 | 1 | 2 |  |  | 52 | 7 |
| *P. raimondii* | OL639020 | 51 | 1 | 3 |  |  | 55 | 8 |
| *P. raimondii* | OL639021 | 50 | 1 | 3 |  |  | 54 | 8 |
| *P. santosii* | OL639022 | 44 | 1 | 3 |  | 1 | 49 | 7 |

Appendix S6 Dispersed repeats of 17 *Puya* accessions

| Species | GenBank/SRA accession number | Forward repeat | Palindromic repeat | Reverse repeat | Complement repeat |
| --- | --- | --- | --- | --- | --- |
| *P. alpestris* | SRR13700326 | 8 | 13 | 1 |  |
| *P. coerulea* | SRR9846915 | 4 | 4 |  |  |
| *P. ferruginea* | OL639024 | 4 | 13 |  |  |
| *P. goudotiana* | OL639023 | 4 | 12 |  | 1 |
| *P. hamata* | MZ403751 | 4 | 11 |  |  |
| *P. hutchisonii* | OL639026 | 4 | 12 |  |  |
| *P. hutchisonii* | SRR10023782 | 4 | 12 |  |  |
| *P. laxa* | SRR13700325 | 4 | 12 |  |  |
| *P. macropoda* | OL639027 | 4 | 12 |  |  |
| *P. macropoda* | OL639028 | 4 | 12 |  |  |
| *P. macrura* | OL639025 | 4 | 12 |  |  |
| *P. macrura* | OL639019 | 4 | 12 |  |  |
| *P. mirabilis* | NC_045380.1 | 4 | 12 |  | 1 |
| *P. nitida* | OL639018 | 5 | 13 |  |  |
| *P. raimondii* | OL639020 | 4 | 12 |  |  |
| *P. raimondii* | OL639021 | 4 | 12 |  |  |
| *P. santosii* | OL639022 | 4 | 12 |  |  |

Appendix S7 The ratio of nonsynonymous and synonymous of 87 protein-coding genes in 17 *Puya* plastomes

| Gene | dS | dN/dS | Gene | dS | dN/dS | Gene | dS | dN/dS | Gene | dS | dN/dS |
| --- | --- | --- | --- | --- | --- | --- | --- | --- | --- | --- | --- |
| *accD* | 0.0008 | 0.2290 | *ndhJ* | 0.0006 | 0.2960 | *psbK* | 0.0005 | 0.5259 | *rpoC1* | 0.0006 | 0.2930 |
| *atpA* | 0.0006 | 0.1307 | *ndhK* | 0.0006 | 0.5343 | *psbL* | 0.0000 | 999.0000 | *rpoC2* | 0.0007 | 0.2584 |
| *atpB* | 0.0002 | 0.1212 | *petA* | 0.0003 | 0.4712 | *psbM* | 0.0012 | 0.0001 | *rps11* | 0.0013 | 0.1489 |
| *atpE* | 0.0010 | 0.5016 | *petB* | 0.0004 | 0.0001 | *psbN* |  |  | *rps14* | 0.0000 | 149.3306 |
| *atpF* | 0.0007 | 0.3072 | *petD* | 0.0006 | 0.1293 | *psbT* |  |  | *rps15* | 0.0005 | 0.8217 |
| *atpH* | 0.0007 | 0.0001 | *petG* | 0.0008 | 0.0001 | *psbZ* | 0.0008 | 0.0001 | *rps16* | 0.0007 | 0.4666 |
| *atpI* | 0.0005 | 0.1143 | *petL* | 0.0016 | 0.2737 | *rbcL* | 0.0006 | 0.1850 | *rps18* | 0.0008 | 0.0001 |
| *ccsA* | 0.0006 | 0.6544 | *petN* | 0.0000 | 0.0001 | *rpl12* |  |  | *rps19* | 0.0000 | 999.0000 |
| *cemA* | 0.0002 | 2.5233 | *psaA* | 0.0006 | 0.0909 | *rpl12* |  |  | *rps19* | 0.0009 | 999.0000 |
| *clpP* | 0.0004 | 0.0001 | *psaB* | 0.0010 | 0.0203 | *rpl14* | 0.0000 | 999.0000 | *rps2* | 0.0009 | 0.2401 |
| *infA* | 0.0005 | 0.0001 | *psaC* | 0.0005 | 0.0001 | *rpl16* | 0.0005 | 0.0001 | *rps3* | 0.0008 | 0.0775 |
| *matK* | 0.0006 | 0.6343 | *psaI* |  |  | *rpl2* |  |  | *rps4* | 0.0006 | 0.1290 |
| *ndhA* | 0.0009 | 0.1992 | *psaJ* | 0.0024 | 0.0001 | *rpl2* |  |  | *rps7* | 0.0000 | 999.0000 |
| *ndhB* | 0.0000 | 999.0000 | *psbB* | 0.0003 | 0.0848 | *rpl20* | 0.0005 | 1.5745 | *rps7* | 0.0000 | 26.0646 |
| *ndhB* | 0.0000 | 999.0000 | *psbB* | 0.0003 | 0.0848 | *rpl22* | 0.0010 | 0.3091 | *rps8* | 0.0016 | 0.1932 |
| *ndhC* | 0.0003 | 1.2404 | *psbC* | 0.0003 | 0.1269 | *rpl23* | 0.0000 | 999.0000 | *ycf1* | 0.0004 | 0.3380 |
| *ndhD* | 0.0011 | 0.1353 | *psbD* | 0.0005 | 0.0001 | *rpl23* | 0.0000 | 999.0000 | *ycf1* | 0.0009 | 0.4468 |
| *ndhE* | 0.0007 | 0.0001 | *psbE* | 0.0004 | 0.0001 | *rpl32* | 0.0000 | 999.0000 | *ycf2* | 0.0001 | 0.4468 |
| *ndhF* | 0.0011 | 0.3103 | *psbF* |  |  | *rpl33* |  |  | *ycf2* | 0.0001 | 0.4265 |
| *ndhG* | 0.0008 | 0.2736 | *psbH* | 0.0007 | 0.2717 | *rpl36* | 0.0017 | 0.2038 | *ycf3* | 0.0002 | 0.0001 |
| *ndhH* | 0.0011 | 0.2375 | *psbI* | 0.0009 | 0.0001 | *rpoA* | 0.0003 | 1.2166 | *ycf4* | 0.0007 | 0.4102 |
| *ndhI* | 0.0010 | 0.0759 | *psbJ* | 0.0012 | 0.0001 | *rpoB* | 0.0006 | 0.2475 |  |  |  |
